# Early Postoperative Endoscopic Findings Following Oversewn Repair of Prepyloric Ulcer Perforation: A Case Report

**DOI:** 10.7759/cureus.110812

**Published:** 2026-06-14

**Authors:** Zain Bukhari, Manoj Nair, Ponnuthurai Pratheepan

**Affiliations:** 1 Department of Surgery, North Middlesex University Hospital, London, GBR; 2 Department of General Surgery, North Middlesex University Hospital, Royal Free London NHS Foundation Trust, University College London (UCL) Medical School, St George’s University Medical School, London, GBR; 3 Department of Colorectal Surgery, North Middlesex University Hospital NHS Trust, London, GBR

**Keywords:** anemia, oversewn repair, peptic ulcer perforation (pulp), postoperative complications, suture line healing, upper gastrointestinal endoscopy

## Abstract

Early postoperative endoscopic evaluation after repair of a perforated gastroduodenal ulcer is rarely performed due to concerns regarding disruption of the surgical closure. We report a patient who underwent primary sutured repair with omental reinforcement for a prepyloric ulcer perforation and subsequently developed postoperative anemia without an identifiable source of bleeding. Initial imaging, including computed tomography angiography, revealed no evidence of active arterial bleeding, prompting a carefully planned endoscopic evaluation in the third postoperative week. Endoscopy demonstrated an intact suture line with healthy granulation tissue and visible intraluminal Vicryl sutures, without evidence of bleeding or dehiscence. This case highlights that early postoperative endoscopic assessment can be safely performed when clinically indicated and provides valuable insight into the intraluminal healing process following oversewn ulcer perforation repair.

## Introduction

Peptic ulcer perforation is a well-recognized surgical emergency and remains a significant cause of morbidity worldwide [1]. Standard operative management involves primary closure of the defect, often reinforced with an omental patch. Although postoperative endoscopy is routinely used to assess ulcer healing, it is typically delayed by several weeks due to concerns that insufflation or instrument manipulation may compromise the repair [2]. As a result, early intraluminal views following surgical closure are seldom reported, and little is known about the appearance of the suture line during the initial healing phase. This case highlights a rare opportunity to evaluate the postoperative anatomy endoscopically within two weeks after repair, prompted by an unexplained fall in hemoglobin. The findings contribute to understanding the safety and potential diagnostic value of early endoscopy when clinical indications justify its use.

## Case presentation

A 60-year-old woman with a history of epilepsy on regular antiepileptic (sodium valproate) therapy presented with sudden-onset upper abdominal pain, repeated vomiting, and absence of bowel movements for five days. There was no history of nonsteroidal anti-inflammatory drug use, gastrointestinal bleeding, or recent weight loss. On presentation, she was hemodynamically stable but in significant discomfort. Abdominal examination revealed generalized tenderness with guarding, raising suspicion of an intra-abdominal perforation. Blood investigations showed inflammatory markers consistent with intra-abdominal sepsis, while hemoglobin at presentation was in the lower normal range. Liver and renal function tests were within normal limits. Computed tomography of the abdomen and pelvis revealed free intraperitoneal air, localized fluid, and a clear perforation on the anterior wall of the prepyloric stomach, with no other associated pathology (Figure 1).

**Figure 1:**
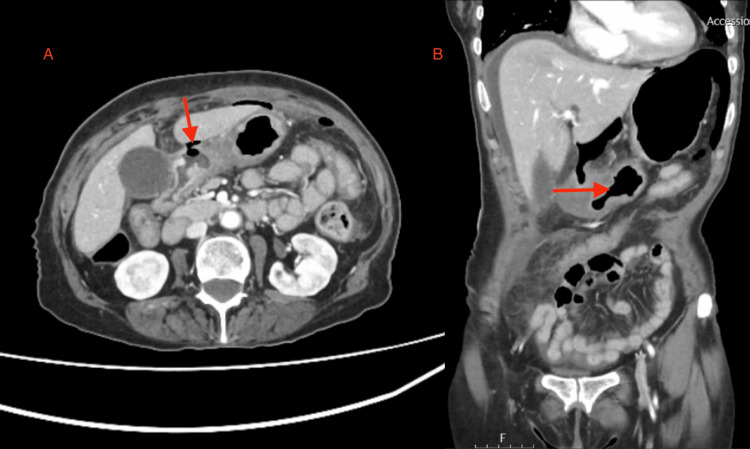
Contrast-enhanced computed tomography of the abdomen demonstrating gastric perforation (A) Axial view and (B) coronal view demonstrating free intraperitoneal air adjacent to the distal stomach (red arrows), consistent with gastric perforation in the prepyloric region

The patient underwent emergency midline laparotomy, during which a 1-cm perforation was identified on the anterior prepyloric region. The defect was closed using interrupted polyglactin 910 (Vicryl) sutures and reinforced with an omental patch. Extensive peritoneal lavage was performed, and intra-abdominal drains were placed.

The patient initially stabilized postoperatively; however, by postoperative days 10-14, she developed a progressive decline in hemoglobin requiring packed red blood cell transfusion. She remained hemodynamically stable with no overt signs of gastrointestinal bleeding. Repeat computed tomography with angiographic protocol demonstrated no active arterial blush but suggested a possible low-pressure venous ooze at the operative site. Given the diagnostic uncertainty and persistent anemia, an early oesophagogastroduodenoscopy was performed at three weeks after surgery by an experienced endoscopist using minimal insufflation.

Endoscopic evaluation revealed no intraluminal blood or clot, an intact suture line, a well-healed ulcer base, and visible intraluminal Vicryl sutures without evidence of disruption or leak. Healthy granulation tissue was noted surrounding the repair site. Systematic examination also demonstrated normal mucosa in the first and second parts of the duodenum (Figure 2).

**Figure 2:**
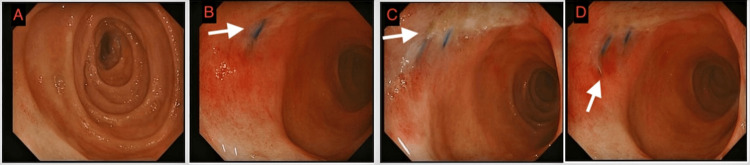
Endoscopic evaluation following repair of prepyloric ulcer perforation (A) Endoscopic view of the second part of the duodenum showing normal mucosa without evidence of bleeding or pathology. (B-D) Endoscopic images of the first part of the duodenum demonstrating intact mucosa with visible intraluminal Vicryl sutures (arrows) and surrounding mild inflammatory changes, without evidence of active bleeding or dehiscence

Following endoscopic assessment, the patient’s hemoglobin stabilized without further transfusion requirements. Oral intake was gradually resumed, and she was discharged in stable condition with continuation of proton pump inhibitor therapy. On follow-up, she remained asymptomatic with no recurrence of abdominal pain or bleeding. Laboratory investigations at presentation and during follow-up are summarized in Table 1.

**Table 1:** Laboratory investigations at presentation and follow-up

Parameter	Result (initial)	Result (follow-up)	Reference range
Hemoglobin (g/L)	79	86	120-160
White cell count (×10⁹/L)	23.55	19.75	4-11
Platelet count (×10⁹/L)	446	453	150-400
Hematocrit	0.234	0.257	0.36-0.46
Red cell count (×10¹²/L)	2.35	2.58	4.0-5.2
Mean corpuscular volume (fL)	99.6	99.6	80-96
Mean corpuscular hemoglobin (pg)	33.6	33.3	27-33
Mean corpuscular hemoglobin concentration (g/L)	338	335	320-360
Neutrophils (×10⁹/L)	21.52	18.82	2-7.5
Lymphocytes (×10⁹/L)	1.06	0.49	1-4
Prothrombin time (seconds)	12.0	13.0	11-13.5
International normalized ratio	1.16	1.27	0.8-1.2
Activated partial thromboplastin time (seconds)	50	49	25-35
Activated partial thromboplastin time ratio	1.7	1.6	0.8-1.2
Sodium (mmol/L)	143	144	135-145
Potassium (mmol/L)	3.9	3.2	3.5-5.0
Urea (mmol/L)	3.1	4.8	2.5-7.8
Creatinine (µmol/L)	41	62	45-90
Estimated glomerular filtration rate (mL/minute/1.73 m²)	>90	>90	>60
Total bilirubin (µmol/L)	3	4	3-20
Alanine transaminase (U/L)	18	12	7-56
Alkaline phosphatase (U/L)	59	47	44-147
Albumin (g/L)	28	28	35-50
Calcium (mmol/L)	2.14	1.99	2.2-2.6
Adjusted calcium (mmol/L)	2.47	2.32	2.2-2.6
Phosphate (mmol/L)	0.47	1.04	0.8-1.5
C-reactive protein (mg/L)	300.0	169.1	<5

## Discussion

Perforated peptic ulcer disease continues to be an important global surgical emergency, most commonly managed with primary closure and an omental patch reinforced by antimicrobial therapy and acid suppression. Standard guidelines recommend delayed endoscopic evaluation several weeks after surgery to document ulcer healing, confirm eradication of *Helicobacter pylori*, and exclude malignancy, particularly in gastric ulcers [1,2]. Early endoscopy is traditionally avoided due to theoretical concerns that insufflation may increase intraluminal pressure and jeopardize the integrity of the suture line or omental patch.

Evidence regarding the optimal timing and safety of postoperative endoscopy after repair of perforated ulcers remains limited [3]. A recent retrospective analysis of patients undergoing surgical repair demonstrated that follow-up endoscopy performed within six to eight weeks detected persistent ulcers and occasionally identified malignant pathology not recognized at the time of surgery [4]. Earlier work also showed that endoscopic healing at approximately six weeks varied depending on *H. pylori* status, with untreated infection associated with delayed mucosal recovery [5]. These studies support delayed surveillance but do not describe the appearance or safety of endoscopic evaluation within the initial postoperative weeks.

Only a small number of published reports describe early postoperative endoscopy after gastroduodenal ulcer repair, and they primarily focus on complications such as postoperative bleeding or persistent ulceration rather than normal healing. Most assessments are performed after several weeks, and very few provide intraluminal images of the repair site during the early healing phase. Consequently, there is minimal published visual documentation of intraluminal sutures or granulation tissue formation in the early postoperative period. The present case contributes rare photographic evidence demonstrating intact polyglactin sutures and healthy granulation tissue in the third postoperative week.

The decision to perform early endoscopy was based on persistent anemia with equivocal findings on computed tomography. Although computed tomography with an angiographic protocol is sensitive for detecting arterial bleeding, its interpretation in the early postoperative period is complicated by expected surgical changes and by the difficulty of differentiating postoperative inflammation from slow venous oozing. Endoscopy, therefore, offered direct mucosal visualization when imaging alone was insufficient to guide management. The procedure was undertaken with minimal insufflation by an experienced endoscopist, consistent with approaches used in early postoperative endoscopy after other upper gastrointestinal surgeries, in which controlled carbon dioxide insufflation has been associated with reduced tension at repair sites.

This case adds several important insights. First, early postoperative endoscopy may be safe in selected circumstances when strongly indicated and performed with meticulous technique. Second, the intact appearance of the suture line suggests that gastric repairs may tolerate gentle insufflation earlier than conventionally assumed. Third, these findings expand the sparse visual literature on early healing after oversewn gastric perforation, providing clinicians with a reference point for interpreting postoperative anatomy. The marked lymphopenia observed during follow-up, together with hypoalbuminemia, may reflect a combination of postoperative physiological stress and nutritional compromise, both of which can potentially influence immune function and wound healing in critically ill surgical patients. The persistently prolonged activated partial thromboplastin time observed during the patient’s course may represent transient perioperative coagulopathy or treatment-related effects and could potentially have influenced postoperative bleeding risk; however, definitive etiological evaluation was not available from the documented records.CO₂ insufflation was utilized during the procedure, which may have contributed to reduced gastrointestinal distension and procedural safety during early postoperative endoscopic evaluation following ulcer repair.

Limitations include the single-patient nature of this report and the requirement for endoscopists skilled in low-insufflation techniques. Nonetheless, this case strengthens the argument that early endoscopy can be considered when diagnostic uncertainty persists after imaging, and it provides uncommon visual documentation of the early healing process, an area largely absent from existing literature.

## Conclusions

Early postoperative endoscopy may be considered in carefully selected patients following primary repair of a perforated prepyloric ulcer, particularly in the setting of persistent anemia or diagnostic uncertainty when imaging findings are inconclusive. In this case, endoscopy performed by an experienced endoscopist with controlled CO₂ insufflation provided a useful intraluminal assessment, with no evidence of repair-site disruption. Visualization of intact sutures and healthy granulation tissue during the third postoperative week offered reassurance regarding healing and helped exclude active intraluminal bleeding. The case also highlights the potential limitations of computed tomography in identifying subtle postoperative findings and demonstrates the complementary diagnostic role of endoscopy in selected scenarios. However, as these observations are based on a single case, broader conclusions regarding the safety, optimal timing, and general applicability of early postoperative endoscopy after perforated gastric ulcer repair cannot be drawn and require further prospective evaluation.
